# The complete chloroplast genome of *Epimedium trifoliolatobinatum* (Koidz.) Koidz. (Berberidaceae)

**DOI:** 10.1080/23802359.2022.2086079

**Published:** 2022-06-30

**Authors:** Yixin Zhang, Xiang Liu, Cheng Zhang, Chaoqun Xu, Fengmei Suo, Weihan Qin, Guoan Shen, Baolin Guo

**Affiliations:** a Institute of Medicinal Plant Development, Chinese Academy of Medical Science, Peking Union Medical College, Beijing, China; b Chongqing Key Laboratory of Traditional Chinese Medicine Resource, Chongqing Academy of Chinese Materia Medica, Chongqing, China; c College of Life Sciences, Key Laboratory of Biodiversity Science and Ecological Engineering, Ministry of Education, Beijing Normal University, Beijing, China

**Keywords:** Chloroplast genome, *Epimedium trifoliolatobinatum*, Berberidaceae, infrageneric classification, phylogenetic relationship

## Abstract

*Epimedium* L. is an important genus in the family Berberidaceae. *Epimedium trifoliolatobinatum* (Koidz.) Koidz. 1939 is inhabited on the west side of the Shikoku, Japan. In this study, the first complete chloroplast genome of *E. trifoliolatobinatum* was assembled with Illumina paired-end sequencing data, which was 157,272 bp in length with a total GC content of 38.70%. A total of 112 unique genes were annotated, comprising 78 protein-coding genes, 30 tRNA genes, and four rRNA genes. The phylogenetic analysis suggested that *E. trifoliolatobinatum* was sister to *E. koreanum*. The current results provided fundamental information for further conducting molecular systematics and phylogenetic research of *Epimedium* genus.

*Epimedium* L. is an important herbaceous genus belonging to the family Berberidaceae, which is composed of over 60 plant species distributed in disjunctive regions ranging from Africa (Algeria) to East Asia (Stearn 2002; Ying 2002). *Epimedium* plants are important medicinal plants possessing excellent biological activities, such as anti-tumor, regulating bone remodeling and so on (Ma et al. 2011; Fan and Quan 2012). The most recent classification for *Epimedium* genus proposed by Stearn recognized two subgenera, four sections and four series (Stearn 2002), among which, *Epimedium trifoliolatobinatum* (Koidz.) Koidz. 1939 belongs to the section *Macroceras*.

However, the infrageneric classification of *Epimedium* genus remains problematic all along (De Smet et al. 2012). Chloroplast genomes are regarded as an important tool for phylogenetic analysis, owing to special features such as moderate nucleotide substitution rate, highly conservative gene sequence and genome structure, etc. (Zhang and Li 2011). Here, we reported the first complete chloroplast genome of *Epimedium trifoliolatobinatum* (Koidz.) Koidz. 1939, providing fundamental data for dissecting the phylogenetic relationships within the genus *Epimedium*.

*E. trifoliolatobinatum* is distributed at the west side of Shikoku, Japan (Stearn 2002). The sample of *E. trifoliolatobinatum* was collected from Rendai, Kochi city, Kochi Prefecture, Japan (latitude 33.5833 and longitude 133.4912). A specimen was deposited at the Herbarium of the Institute of Medicinal Plant Development (IMPLAD), Beijing, China (http://www.implad.ac.cn/, contact Baolin Guo, blguo@implad.ac.cn) under voucher number B. L. Guo JP04. Genomic DNA was extracted from the silica dried leaves of *E. trifoliolatobinatum* with the modified CTAB method (Doyle and Doyle 1987). The complete *E. trifoliolatobinatum* chloroplast genome was sequenced on Illumina Novaseq 6000 platform (Illumina Inc., San Diego, CA), and the assembly was performed by using GetOrganelle v1.5 (Jin et al. 2018). The gene annotation was conducted through CPGAVAS2 (Shi et al. 2019), and manually corrected.

The size of *E. trifoliolatobinatum* chloroplast genome (MW483095) was 157,272 bp, with a quadripartite structure containing four distinct regions of a large single-copy (LSC, 89,613 bp) region and a small single-copy (SSC, 17,223 bp) region divided by a pair of inverted repeat regions (IR_A_ and IR_B_, 25,218 bp). The overall GC content was 38.78%. The GC content of IR regions was found to be the highest (43.30%), which was 37.25% and 32.79% in the LSC region and SSC region, respectively. The entire *E. trifoliolatobinatum* chloroplast genome was found to encode 112 unique genes, including 78 protein-coding genes, four ribosomal RNA genes, and 30 tRNA genes. Among the 18 intron-containing genes detected are 15 genes containing one intron, and three genes containing two introns.

The complete chloroplast genomes of *E. trifoliolatobinatum* along with other 11 species downloaded from NCBI database were aligned with MAFFT v7 (Katoh et al. 2019), and used for phylogenetic analysis. *Vancouveria hexandra* was selected as the outgroup. The maximum-likelihood (ML) tree (1000 replicates) and Bayesian’s inference (BI) tree (1,000,000 generations) were constructed by using IQ-TREE multicore v 2.0.3 (Minh et al. 2020) and MrBayes 3.2.7 package (Ronquist and Huelsenbeck 2003) (Figure 1), respectively. As a result, the ML and BI phylogenetic trees displayed identical topologies, indicating that *E*. *trifoliolatobinatum* formed a sister relationship with *E. koreanum* (section *Macroceras*).

**Figure 1.**
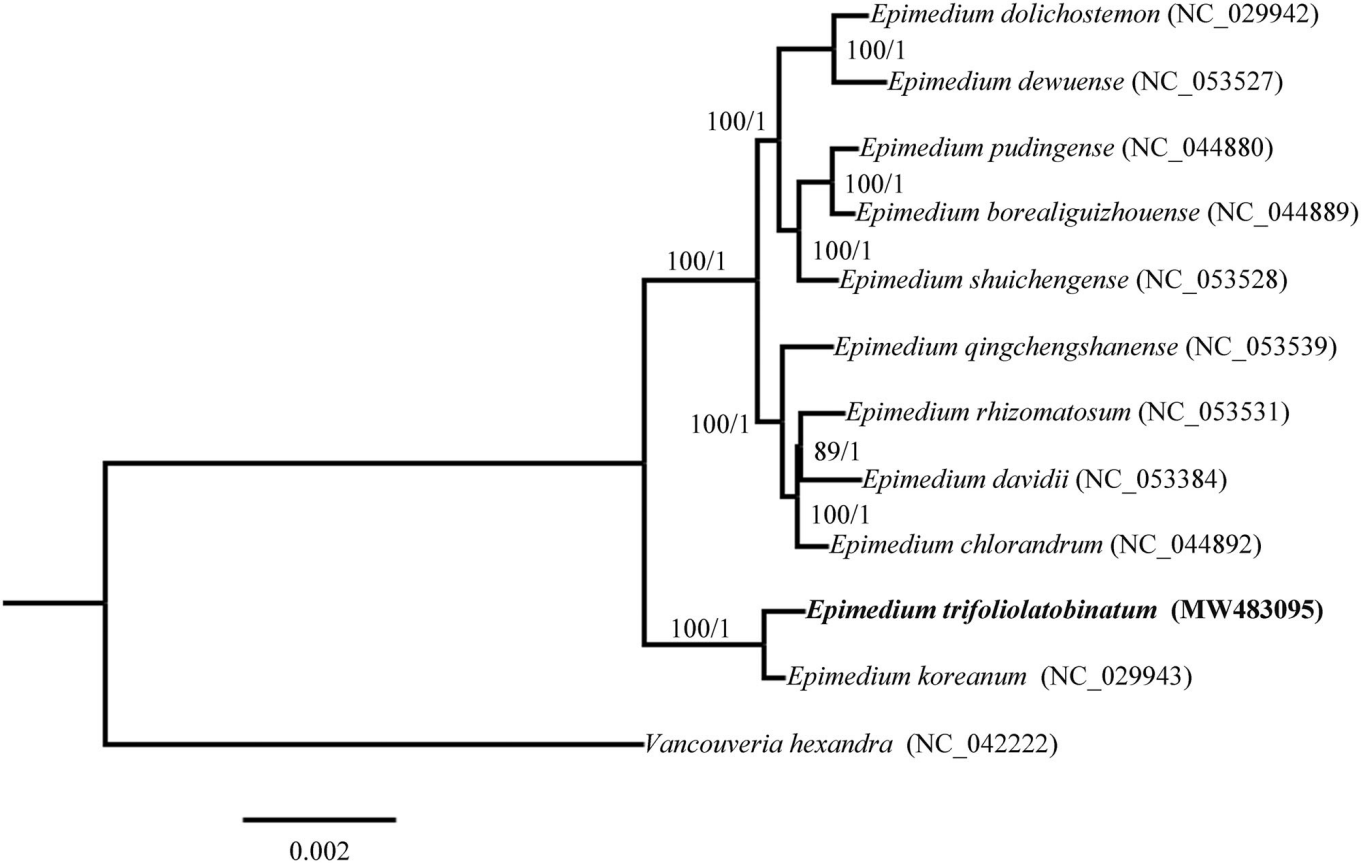
Maximum-likelihood (ML) and Bayesian’s inference (BI) phylogenetic tree based on complete chloroplast genomes of 12 species, with *Vancouveria hexandra* as outgroup. Numbers at the nodes represent maximum-likelihood bootstrap support and Bayesian’s inference posterior probabilities, respectively.

## Data Availability

The genome sequence data that support the findings of this study are openly available in GenBank of NCBI at https://www.ncbi.nlm.nih.gov/ under the accession no. MW483095. The associated numbers are PRJNA763308, SRR16937570, and SAMN21437729, respectively.
